# Tunneling nanotube-mediated intercellular vesicle and protein transfer in the stroma-provided imatinib resistance in chronic myeloid leukemia cells

**DOI:** 10.1038/s41419-019-2045-8

**Published:** 2019-10-28

**Authors:** Marta D. Kolba, Wioleta Dudka, Monika Zaręba-Kozioł, Agata Kominek, Paolo Ronchi, Laura Turos, Piotr Chroscicki, Jakub Wlodarczyk, Yannick Schwab, Agata Klejman, Dominik Cysewski, Katja Srpan, Daniel M. Davis, Katarzyna Piwocka

**Affiliations:** 1 Laboratory of Cytometry, Nencki Institute of Experimental Biology, Polish Academy of Sciences, Warsaw, Poland; 2 Laboratory of Cell Biophysics, Nencki Institute of Experimental Biology, Polish Academy of Sciences, Warsaw, Poland; 3 Electron Microscopy Core Facility, European Molecular Biology Laboratory, Heidelberg, Germany; 4 Cell Biology and Biophysics Unit, European Molecular Biology Laboratory, Heidelberg, Germany; 5 Laboratory of Animal Models, Nencki Institute of Experimental Biology, Polish Academy of Sciences, Warsaw, Poland; 6 Mass Spectrometry Laboratory, Institute of Biochemistry and Biophysics, Polish Academy of Sciences, Warsaw, Poland; 7 Manchester Collaborative Centre for Inflammation Research, University of Manchester, Manchester, UK

**Keywords:** Cancer microenvironment, Mechanisms of disease, Haematological diseases

## Abstract

Intercellular communication within the bone marrow niche significantly promotes leukemogenesis and provides protection of leukemic cells from therapy. Secreted factors, intercellular transfer of mitochondria and the receptor–ligand interactions have been shown as mediators of this protection. Here we report that tunneling nanotubes (TNTs)—long, thin membranous structures, which have been identified as a novel mode of intercellular cross-talk—are formed in the presence of stroma and mediate transfer of cellular vesicles from stroma to leukemic cells. Importantly, transmission of vesicles via TNTs from stromal cells increases resistance of leukemic cells to the tyrosine kinase inhibitor, imatinib. Using correlative light-electron microscopy and electron tomography we show that stromal TNTs contain vesicles, provide membrane continuity with the cell bodies and can be open-ended. Moreover, trans-SILAC studies to reveal the non-autonomous proteome showed that specific sets of proteins are transferred together with cellular vesicles from stromal to leukemic cells, with a potential role in survival and adaptation. Altogether, our findings provide evidence for the biological role of the TNT-mediated vesicle exchange between stromal and leukemic cells, implicating the direct vesicle and protein transfer in the stroma-provided protection of leukemic cells.

## Introduction

Chronic myeloid leukemia (CML) results from a reciprocal chromosomal translocation, which gives rise to a bcr-abl fusion gene^1^. The BCR-ABL1 protein is a constitutively active tyrosine kinase capable of uncontrolled signaling to numerous downstream targets^2^. Since the introduction of imatinib^3,4^, the annual mortality rate has decreased significantly^5^. This therapy is successful in the chronic phase of disease, however most patients either do not achieve a complete cytogenetic response or develop resistance and progress to the blast phase. Importantly, cells that are isolated and cultured in vitro often lose their chemoresistant phenotype, suggesting a cytoprotective role of the bone marrow microenvironment^6^. The stroma-provided protection of CML cells from imatinib treatment has been well documented^7–9^. Additionally, we and others have shown that stromal and leukemic cells interact bidirectionally, to support leukemogenesis^10–12^. Overall, intercellular communication is an important player in the stroma-mediated resistance of CML. However, our understanding of how stromal cells influence leukemia is still incomplete.

Tunneling nanotubes (TNTs) or membrane nanotubes are long, thin membranous structures that connect distant cells, first characterized as open membrane conduits^13^. However, some types of membrane immune synaptic nanotube were also discovered to contain a submicron scale junction, or synaptic connection, enabling transfer of cargo^14–17^. Recent studies using correlative cryo-electron microscopy showed 3D ultrastructure of TNTs in neuronal cells^18^ however most of studies failed to establish a clear structure for TNTs.

TNTs or membrane nanotubes more broadly, have been recognized as a novel mode of intercellular communication^19–21^, enabling transfer of cellular vesicles^13,15^, mitochondria^22–25^, miRNAs^26^, viral particles^17^ and single proteins^27,28^. They have been found in variety of cell types: immune cells^14,15^, neurons^29^, endothelial cells^30,31^ or mesenchymal stromal cells ^32^.

In cancer cells, TNTs formation might correlate with invasion^33,34^. Moreover, TNT-mediated mitochondria transfer has been shown to promote chemoresistance^26,35,36^. Recently, TNTs have been also found in blood cancers and within their microenvironment. Acute myeloid leukemia cells have been shown to form homotypic TNTs^37^ as well as heterotypic ones with bone marrow cells^38^. The latter resulted in transfer of mitochondria toward AML cells, suggesting a survival advantage. Acute lymphoid leukemia cells signal to mesenchymal stromal cells through TNTs, promoting secretion of proleukemic cytokines^39^. Recent studies showed that the HTLV-1 transmission between leukemic cells occurs via TNTs^37^. Together, these studies are consistent with leukemic cells using multiple TNT-dependent processes. Even if TNT-mediated vesicles transfer has been shown in leukemia, the in-depth studies of the functional consequences of cellular vesicles transfer within leukemia microenvironment are lacking. Here, we report evidence for the direct traffic of vesicles via TNTs from stromal to CML cells providing protection against imatinib-induced apoptosis. Specific functional sets of proteins with possible roles in survival and adaptation were directly transferred together with vesicles. Moreover, using CLEM tomography we showed the ultrastructure of stromal TNTs. Altogether, these studies indicate TNT-mediated vesicle and protein transfer as important players in the stroma-provided protection of leukemia.

## Materials and methods

### Cell lines and reagents

K562 (ATCC#CCL-243) and HS-5 (ATCC#CRL-11882) cell lines were cultured in RPMI medium that was supplemented with 10% fetal bovine serum (FBS), 1% l-glutamine, and 1% penicillin streptomycin. Both cell lines were recently been authenticated by the ATCC Cell Line Authentication Service, using Short Tandem Repeat (STR) analysis, and underwent a regular screen for *Mycoplasma* contamination by RT-PCR. The K562-GFP cell line was established by Dr. M. Kusio-Kobiałka. Imatinib was a generous gift from the Pharmaceutical Research Institute (Warsaw) and used at concentrations of 0.5, 1, and 2 µM.

### Co-culture system and flow cytometry measurements

#### Exchange of cargo between cells

Donor cells were labelled with DiD (catalog no. V22887, ThermoFisher Scientific), 1.5 μl/1 ml of cell culture medium for 15 min at 37 °C, washed and plated in fresh cell culture medium for an additional 16 h. To analyze mitochondria transfer, HS-5 cells were transduced with rLV.EF1.AcGFP1-mito-9 lentiviral vector (TaKaRa) for stable mitochondria labelling. Afterward, cells were seeded in co-culture with acceptor cells in 12-well cell culture plates (1 × 10^5^ HS-5 cells plus 0.8 × 10^5^ K562 wt or K562-GFP cells) to reach a 1:1 ratio after 24 h. For flow cytometry BD LSRFortessa cytometer (Becton Dickinson Poland) was used, followed by data analysis using Diva and FlowJo software.

#### Transwell and CM controls

To physically separate donor and acceptor cells in co-culture, HS-5 and K562 cells were plated in the lower and upper chambers of a transwell system (ThinCert, Greiner Bio-One), 1 µM pores, 2 × 10^6^ pores/cm^2^, for 24 h. As a control for the conditioned media (CM), donor cells were labeled as described above. After 24 h, the supernatant was collected, centrifuged to remove cells and cellular debris, and added to acceptor cells in 12-well culture plates. After another 24 h, acceptor cells underwent flow cytometry analysis.

#### Flow cytometry measurement of apoptotic cells

Co-cultures of DiD-labeled HS-5 cells with K562 GFP cells were untreated or treated with imatinib for 24 h and stained with AnnexinV-PE and 7-AAD (catalog no. 559763, BD Pharmingen) according to the manufacturer’s instructions. DiD+ and DiD− acceptor cells were separated by gating and analyzed for apoptosis. To study caspase activation, cells were labeled with Violet Live Cell Caspase Probe (catalog no. 565521, BD Pharmingen) according to the manufacturer’s instructions and 7-AAD for live cell discrimination. DiD+ and DiD- acceptor cells were separated by gating, and the percentage of cells with active caspases was calculated. For flow cytometry BD LSRFortessa cytometer was used, followed by data analysis using Diva and FlowJo software.

### Fluorescent imaging and live cell microscopy

#### Immunocytochemistry and immunofluorescence

Cells were plated on poly-l-lysine-coated coverslips, fixed with 4% paraformaldehyde, permeabilized with 0.1% Triton X-100, blocked with 5% FBS and incubated with antibodies and fluorescent stains. Phalloidin (ThermoFisher Scientific) was used for actin staining, DAPI (catalog no. D9542, Sigma-Aldrich) was used for nuclear labeling. Microtubules were labeled with monoclonal anti-β-tubulin antibody (catalog no. T0198, Sigma-Aldrich), MyoVa antibody, (catalog no. 3402S, ThermoFisher Scientific), MyoVI antibody (catalog no. 25–6791, Proteus), and MyoVIIa antibody (catalog no. 25–6790, Proteus). Mitochondria and cellular vesicles were labeled with 250 nM MitoTracker Deep Red (catalog no. M22426, ThermoFisher Scientific) or DiD, respectively. Images were acquired using a Zeiss LSM 780 microscope with a 63× objective and further processed using ImageJ and Imaris software.

#### Tunneling nanotube imaging in living cells

HS-5 and K562 cells expressing GFP were plated on poly-l-lysine coated Lab-Tek Chamber Slides (ThermoFisher Scientific). Plasma membranes were labeled with Wheat Germ Agglutinin (WGA) conjugates: WGA-AF 647 (catalog no. W32466, ThermoFisher Scientific) or WGA-AF 488 (catalog no. W11261, ThermoFisher Scientific). Images were acquired using an SP8 Leica microscope with a ×63 or ×100 objective. For TNTs quantification ten fields (155 μm × 155 μm) with z-stacks that covered the majority of the cell volume were acquired using SP8 Leica microscope with a 63× or 100× objective. The data were manually analyzed using ImageJ software with the Cell Counter plugin.

#### Plasma membrane contribution

K562 cells were transfected by nucleofection (Amaxa Nucleofector Technology, Lonza AG) using a GPI-GFP plasmid (kind gift from D. Davis). After 24 h cells were sorted based on GFP fluorescence and co-cultured with HS-5 cells on Lab-Tek Chamber Slides (ThermoFisher Scientific) for 48 h. Additionally, both cell types were stained with WGA-Alexa Fluor 647 (ThermoFisher Scientific). Images were acquired using an SP8 Leica microscope with a ×63 objective and analyzed using ImageJ software.

### Electron microscopy

#### Scanning electron microscopy

Cells were plated on poly-l-lysine-coated coverslips. After 48 h, cells were fixed with 2.5% glutaraldehyde and 2% PFA, followed by washing with PBS, water and dehydration by subsequent incubation in an ascending series of ethanol concentrations (50%, 70%, 96%, and 99.9%, 10 min each). Then samples were subjected to critical-point drying, gold-coated, and imaged on 3View using Zeiss Sigma VP SEM column. The secondary electron signal was used to obtain an image.

#### Transmission electron microscopy and CLEM tomography

Monolayers of cells were seeded on IBIDI-gridded, glass window, poly-l-lysine-coated plates (Grid-500 Glass Bottom μ-Dish, 35 mm (81168, Ibidi). After 24 h, cells were labeled with WGA AF-647 and fixed in 2.5% glutaraldehyde and 2% PFA in cacodylate buffer. Light microscopy images were acquired using Olympus IX81 widefield microscope (Olympus Germany) with CellR software. Cells were then washed with cacodylate, contrasted in 1% osmium and 1% uranyl acetate (UA), dehydrated in ethanol, and embedded in Epon resin. The fixation, contrasting, and dehydration steps were performed in a PELCO Biowave Pro microwave^40^. 50 serial sections (300 nm thick) were collected on formvar-coated slot grids, 15 nm gold beads were added as fiducial for tomogram reconstruction, and then sections were poststained in 2% UA and lead citrate. Regions of interest (ROI) were imaged using a FEI Tecnai F30 (tomography) electron microscope (300 kV). Tomograms were acquired using combination of Tecnai FEI and SeriaEM softwares. Pixel size 26nm, magnification 4700x, series of images was taken between −60 and +60 degree using double tilt grid holder. Images were acquired with a OneView Camera (Gatan). Tomogram reconstruction processing was performed using the IMOD software package^41^.

### M-Sec silencing

The MISSION® TRC shRNA bacterial stock containing shRNA plasmids targeting M-Sec (TNFAIP2) (Sigma-Aldrich) have been streaked onto a plate and plasmids were isolated by use of Endo Free Maxi Prep kit (Qiagen) after single colony inoculation and overnight culture. Isolated plasmids were verified by restriction enzyme analysis. For viral vector production the Human Embryonic Kidney cells (HEK 293T) cultured in low glucose DMEM (catalog no. 6046, Sigma-Aldrich), supplemented with 10% FBS and 1% penicylin/streptomycin were transiently co-transfected with 3 plasmids containing elements necessary for lentiviral vector production (pVSV—pseudotyping plasmid encoding the vesicular stomatitis virus envelope; p∆8.2—encapsidation plasmid and pMISSION® TRC shRNA plasmid containing the shRNA construct). The polyethylenimine PEI (MW ~ 25 000; Warrington) transfection method was used. Twenty-four hours post transfection the target K562 cells were added and viral infection was performed by 72 h co-culturing of packaging cells and target cells in presence of Polybren (Sigma-Aldrich), final concentration 10 μg per ml. After infection the non-adherent K562 cells were gently removed, centrifuged and resuspended in RPMI medium with 1 µg/ml selection antibiotic Puromycin (Sigma-Aldrich). M-Sec protein level was estimated by Western Blotting using antibody anti-TNFAIP2 alpha (M-Sec) (catalog no. sc-28318, Santa Cruz).

### Trans-SILAC

#### Cell labeling with heavy isotopologues of lysine and arginine

The SILAC medium was supplemented with 10% of dialyzed FBS, 1% Pen/Strep, 0.274 mM l-lysine, and 1.15 mM l-arginine and filtered (0.22 µM pores). Donor cells were labeled with heavy isotopologues of lysine and arginine: l-lysine:2HCL (13C6, 99%; 15N2, 99%) and l-arginine: HCL (13C6, 99%; 15N4, 99%; Cambridge Isotope Laboratories, Sigma-Aldrich). Cells were maintained in the appropriate medium for 9 days to enable complete labeling of the proteome. The medium was changed every 2–3 days. On day 8, donor cells were labeled with DiD as described above. This experiment was performed in duplicate.

#### Co-cultures and cell sorting

Twenty-four hour co-cultures were sorted using a BD FACSAria sorter (Becton Dickinson Poland). GFP fluorescence was excited with a 488-nm laser, and emission was detected with a 530/30 filter. DiD fluorescence was excited with a 633-nm laser, and emission was detected with a 660/20 filter. Donor and acceptor cells were separately gated, followed by sorting of acceptor cells into two subpopulations: positive and negative for transferred vesicles (DiD+ vs DiD-). Cell pellets were immediately frozen in liquid nitrogen and stored at −80 °C until further sample preparation.

#### Sample preparation

Cell pellets were lysed in 25 mM ammonium carbonate and 0.1% Rapigest (pH 8.2). Protein lysate (10 μg) was digested with Trypsin Gold, reduced with 5 mM tris(2-carboxyethyl) phosphine (TCEP), and blocked with 5 mM iodoacetamide. DID+ and DID− samples were diluted with 4× Laemmli buffer, boiled for 5 min at 95 °C, and loaded onto a sodium dodecyl sulfate-polyacrylamide electrophoresis gel. Lanes were cut into six pieces, and proteins were digested using trypsin according to standard protocols^42^. Peptides were extracted, purified by styrenedivinylbenzene reverse-phase sulfonate (SDB-RP); also known as mixed mode chromatography stage tips^43^, and stored at 4 °C prior to MS analysis.

#### LC-MS/MS analysis

Mass spectrometry analysis was performed using an EASY nLC 1000 coupled to a Q-Exactive Plus mass spectrometer (ThermoFisher Scientific). Peptides were separated by a 180-min linear gradient of 95% solution A (0.1% formic acid in water) to 35% solution B (acetonitrile and 0.1% formic acid). The measurement of each sample was preceded by three washing runs to avoid cross-contamination. The mass spectrometer was operated in the data-dependent MS-MS2 mode. Data were acquired in the *m*/*z* range of 300–1750 at a nominal resolution of 70,000.

Data were analyzed using the Max-Quant 1.5.3.12 platform, with the reference human proteome database from UniProt. False discovery rates of protein and peptide-spectrum matches (PSM) levels were estimated using the target-decoy approach at 0.01% (protein FDR) and 0.01% (PSM FDR), respectively. The minimal peptide length was set to 7 amino acids, and carbamidomethyolation at cysteine residues was considered a fixed modification. Oxidation (M) and Acetyl (Protein N-term) were included as variable modifications. Only proteins that were represented by at least two unique peptides in two biological replicates are shown and were further considered. The data analysis was performed using MaxQuant software and the MaxLFQ algorithm (Supplementary Table 1). Lists of proteins were analyzed using the Panther application for GeneOntology software^44^, STRING-confidence view^45^, and Venny 2.1 (http://bioinfogp.cnb.csic.es/tools/venny/index.html). Additionally, lists of proteins were grouped according to their molecular weights based on the UniProt database. The mass spectrometry data from this publication have been deposited to the ProteomeXchange Consortium via the PRIDE [https://www.ebi.ac.uk/pride] partner repository with the dataset identifier PXD013504.

### Statistical analysis

All of the experiments were performed in at least three independent biological repetitions, trans-SILAC has been done in two biological replicates. All of the data are presented as mean ± SEM. Student’s *t*-test with Welch correction was used to test differences between two conditions. Values of *p* < 0.05 were considered statistically significant. Microsoft Excel and GraphPad Prism software were used for the data analysis. *p < 0.05, **p < 0.01, ***p < 0.001.

## Results

### Tunneling nanotubes are formed between CML and stromal cells

**Fig. 1:**
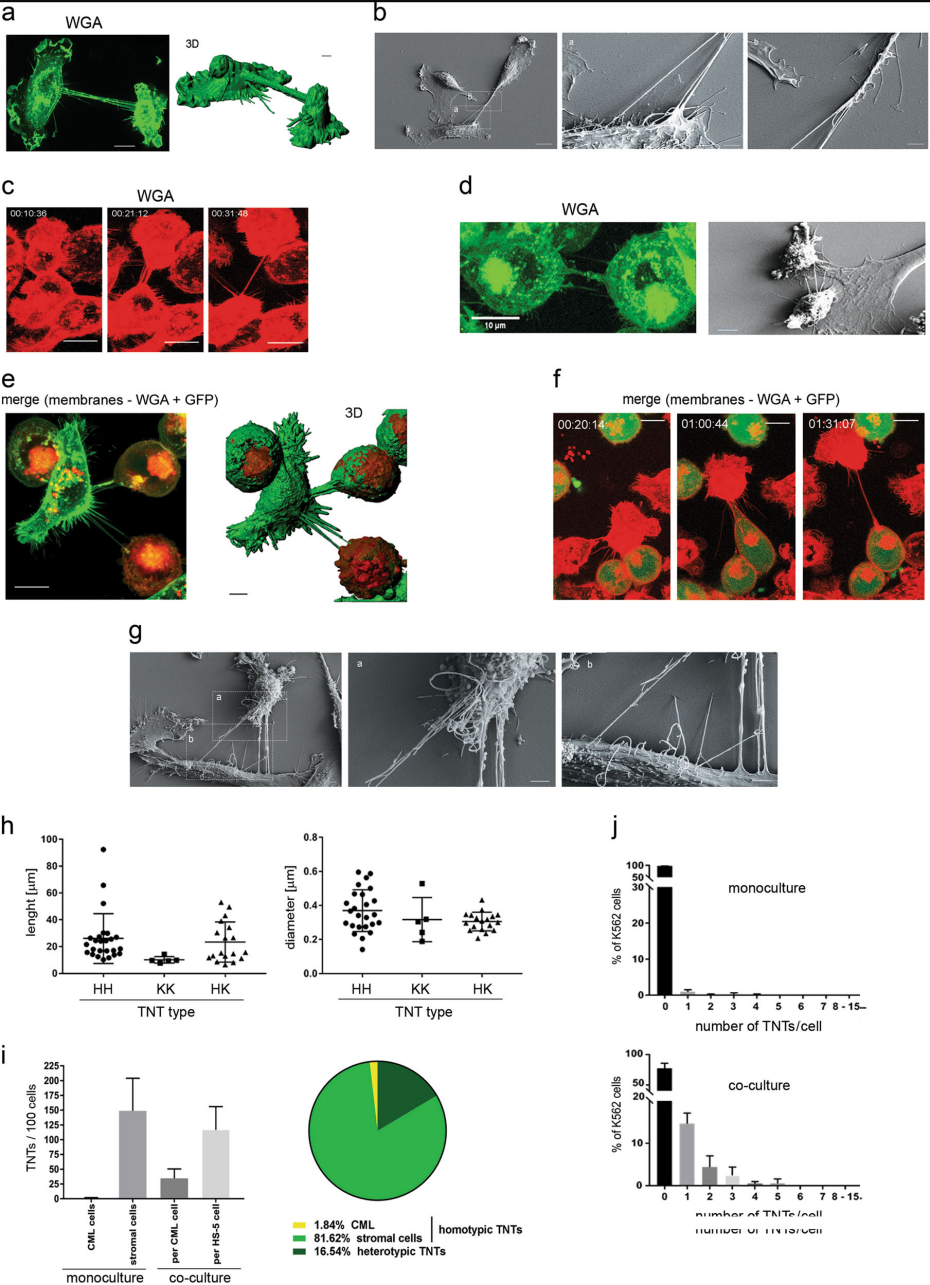
Formation of tunneling nanotubes (TNTs) between leukemic cells and stromal cells. **a** Representative image of TNTs that interconnected stromal cells (left) and 3D reconstruction (right). Cell membranes were stained with WGA-Alexa Fluor 488. Scale bar = 4 μm. **b** Scanning electron microscopy image of TNTs formed between stromal cells. Zoomed images show a TNT end that protruded from the cell body and a bifurcation of the TNT. Scale bars: 10 μm (left image), 2 μm (middle and right images). **c** Selected frames from a time-lapse experiment (h:min:sec) that presents TNT formation between stromal cells. Cell membranes were stained with WGA-Alexa Fluor 647. **d** Representative images showing a TNT formed between leukemic cells visible after cell membranes staining with WGA-Alexa Fluor 488 (left) and after scanning electron microscopy (right). Scale bar = 10 μm. **e** Representative image of heterotypic TNTs that interconnected one stromal cell and two CML cells (left) and 3D reconstruction (right). CML cells were labeled with DiD (red) and all cell membranes were stained with WGA-Alexa Fluor 488 (green) directly before imaging. Scale bar = 4 μm. **f** Selected frames from a time-lapse experiment (h:min:sec) that presents TNT formation between stromal and CML cells. Red indicates all cell membranes that were stained with WGA-Alexa Fluor 647. Green indicates CML cells that expressed cytoplasmic GFP. Scale bars = 10 μm **g** Scanning electron microscopy image of TNTs formed between stromal cells and CML cells. Zoomed images show that TNT ends protruded from cell bodies of stromal and CML cells. Scale bars = 2 μm. **h** Diameters and lengths of homo- and heterotypic TNTs that were measured in living cells by confocal microscopy. Homotypic TNT formed by stromal cells (HH) or leukemic cells (KK) as well as heterotypic TNTs (HK) were measured. **i** Average numbers of TNTs per 100 cells quantified in mono- and co-culture set-ups by confocal microscopy after 24 h of cell culture. The graph (left) shows that stromal cells had a propensity to form TNTs, whereas CML cells were prone to form TNTs only upon co-culture with stromal cells. Prevalence of homo- and heterotypic TNTs in a co-culture set-up, quantified by confocal microscopy. The diagram (right) shows that the majority of TNTs that were found in co-cultures were homotypic TNTs that interconnected stromal cells. Heterotypic TNTs constituted a considerable portion of overall TNTs. CML cells in co-culture were significantly more involved in TNT formation with stromal cells than in TNT formation with other CML cells. **j** Percentage of CML cells that exhibited a given number of TNTs in a monoculture (upper) and co-culture (lower) set-up

To investigate the formation and function of TNTs within the leukemia microenvironment, we first set out to test whether or not HS-5 cells, a model bone marrow stromal cell line^8,46^ and K562 chronic myeloid leukemia cells, are able to form intercellular connections with characteristics of TNTs. Using confocal microscopy followed by 3D reconstruction (Fig. 1a) and scanning electron microscopy (SEM) (Fig. 1b) we confirmed the efficient formation of TNT connecting two distant cells. We found that TNTs are membrane structures which do not touch the substratum (Fig. 1a). All TNTs contained F-actin, whereas tubulin was present only in some (Supplementary Fig. 1). The time-lapse studies of viable cells revealed that stromal TNTs were formed within minutes after direct cell-cell contact followed by cell dislodgement (Fig. 1c, Supplementary Movie 1). In contrast to stromal TNTs, homotypic TNTs formed between CML cells were very rare and relatively short, however they also displayed features representative for TNTs (Fig. 1d).

To examine the formation of heterotypic TNTs, GFP-positive K562 cells were co-cultured with HS-5 stromal cells. Staining of membranes in living cells revealed the presence of structures with TNT features that connected distant cells of two types (Fig. 1e, g). Heterotypic TNTs did not touch the substratum (Fig. 1e, g) and were formed also by cell dislodgement (Fig. 1f, Supplementary Movie 2). In living cells, the average length and diameter of heterotypic TNTs did not differ significantly from homotypic stromal TNTs (Fig. 1 h).

To assess whether plasma membranes of both cell types participate in the formation of heterotypic TNTs, CML cells expressed GFP with a GPI tag to track plasma membrane, in addition to staining of all plasma membranes with WGA-Alexa Fluor 647. We found that membranes from either only one type or originated from both cell types build heterotypic TNTs (Supplementary Fig. 2a), and the length of TNTs did not depend on the plasma membrane origin (Supplementary Fig. 2b).

Calculation of the average number of TNTs per 100 cells (TNT index) showed that only 1.0 ± 0.6 TNTs among 100 CML cells were found at a given time, in comparison to 150.0 ± 32.0 TNTs formed by 100 stromal cells (Fig. 1i, left panel). Upon contact with stroma, CML cells formed more TNTs (TNT index increased up to 35.0 ± 6.0) (Fig. 1i, left panel). The heterotypic TNTs generally accounted for 16.5% ± 3.5% of all TNTs, whereas homotypic CML TNTs constituted only 1.8% ± 1.0% of the overall number (Fig. 1i, middle panel). The presence of stromal component increased leukemic TNTs, increasing not only TNT index (Fig. 1i) but also number of nanotubes per cell (Fig. 1j). Altogether, our analysis revealed that heterotypic TNTs are formed between stromal and leukemic cells and the stroma component stimulates formation of TNTs by leukemia.

### CLEM analysis of TNT ultrastructure of stromal cells

**Fig. 2:**
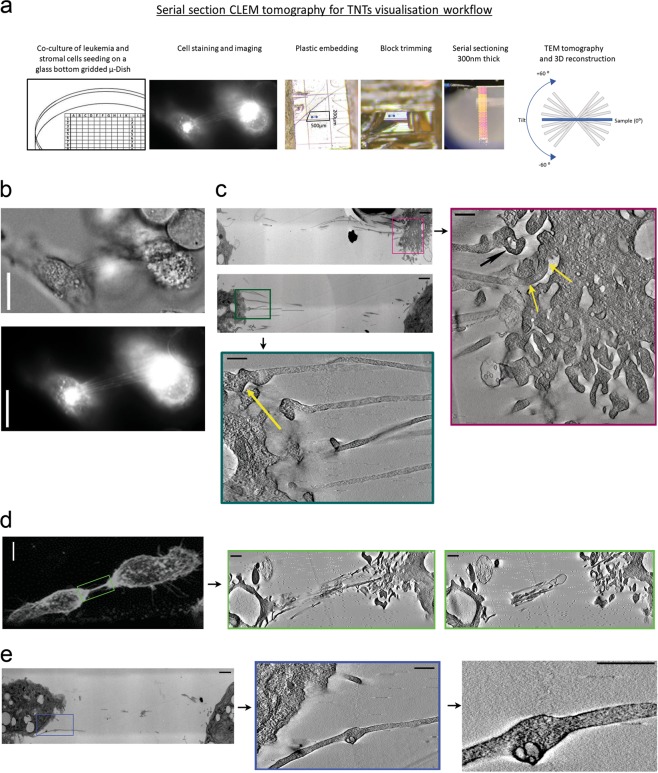
Ultrastructure of tunneling nanotubes (TNTs). **a** The workflow of serial section CLEM tomography used to visualize 3D ultrastructure of stromal TNTs. **b** Correlative light and **c** electron microscopy (CLEM) of the same area from 300 nm sections were imaged by bright field b (upper) and fluorescence microscopy (lower; WGA—stained). View of corresponding tomography stack visible of marked ROI (purple and green frames) showing the presence of TNTs with open ends providing continuity with the cell body (yellow arrows). Possibly closed TNT is indicated with black arrow. Scale bar = 20 μm (**b**), 2 μm (**c**—upper left), 500 nm (**c**—bottom left and right). **d** Fluorescence image of one thick TNT (left) and tomography of ROI (green frame) showing the bunches of single TNTs. Scale bar = 2 μm (left image), 500 nm (middle and right images). **e** Transmission electron microscopy (left) of the TNT with visible bulge (left, blue frame) with corresponding electron tomography view show cellular vesicles present inside a TNT. Scale bar = 500 nm

Having shown that heterotypic TNTs formed between stromal and CML cells resemble those between stromal cells, according to similar size, structure and mechanism of formation, we went on to examine their ultrastructure. To assess the 3D ultrastructure TNTs we used CLEM and electron tomography followed by 3D reconstruction (Fig. 2a). Single TNTs were observed already upon resolution of fluorescence microscopy (Fig. 2b). EM tomography confirmed that single TNT was a tube, continuous with the plasma membrane of the cell body (Fig. 2b, c). The thickness of the TNTs was remarkably consistent (~200 nm). However, apart from single TNTs described above, we also observed bunches of multiple TNTs (Fig. 2d), which were similar to those described in neurons^18^, indicating that both forms are possible within the leukemic stroma. Importantly, the open-ended TNTs were found, confirming possibility of cargo transfer between two distant cells (Fig. 2c, yellow arrows), however also possibly close-ended TNTs were observed (Fig. 2c, black arrow). Membrane vesicles with an average diameter corresponding to the typical size of cellular vesicles (111 ± 33 nm) were present inside TNT lumen (Fig. 2e). Typically, nanotube bulges were seen in places containing vesicles (Fig. 2e). Together with the presence of structures resembling actin filaments that were observed in TNT containing vesicles (Fig. 2e, right panel), these are consistent with cellular vesicles being trafficked via TNTs.

### Cellular vesicles are transferred between stromal and CML cells in TNT-promoting conditions

**Fig. 3:**
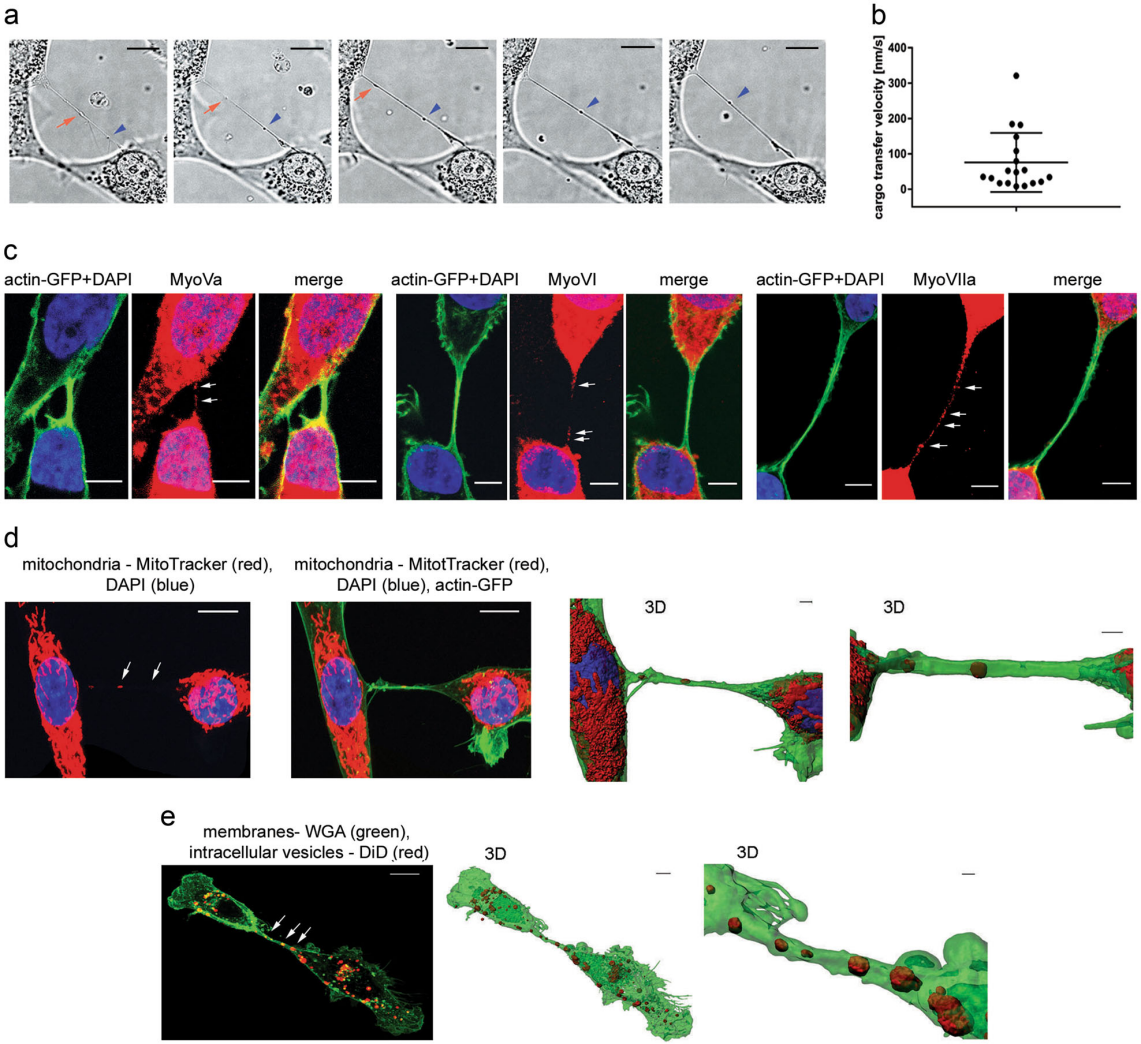
Visualization of cargo transfer mediated by tunneling nanotubes (TNTs) formed between stromal cells. **a** Frames from a time-lapse experiment that present the transport of two bulges along a TNT. Scale bar = 4 μm. **b** Velocity of cargo transfer in the time-lapse experiments (*n* = 18 events). **c** Representative images that show the presence of molecular motors inside TNTs. Green indicates the actin cytoskeleton. Blue indicates nuclei. Red indicates antibody-stained myosin Va (left), myosin VI (middle), and myosin VIIa (right). Scale bar = 5 μm. Single channels and a merged image are shown. **d** Representative images that show the presence of mitochondria inside TNTs (left panels). Green indicates actin. Blue indicates nuclei. Red indicates mitochondria that were stained with MitoTracker Deep Red. The 3D reconstruction shows in detail mitochondria that were present within the lumen of the TNT (right panels). Scale bar = 4 μm. **e** Representative image that shows the presence of cellular vesicles inside TNTs (left panels). Green indicates cell membranes that were stained with WGA-Alexa Fluor 488. Red indicates cytoplasmic vesicles that were stained with DiD. The 3D reconstruction shows in detail cytoplasmic vesicles that were present within the lumen of the TNT (right panels). Scale bar = 4 μm

To dissect the process of vesicular transfer between cells, we quantified the movement of bulges along TNTs (Figs. 3a, b, Supplementary Movie S3). As the speed movement was similar to that reported for myosin-driven movement^47,48^, the presence of myosins was assessed. MyoVa, MyoVI, and MyoVIIa were detected along F-actin containing TNTs (Fig. 3c), suggesting their possible involvement. In order to visualize myosins within thin TNT, the signal had to be increased resulting in overexposure of cellular fluorescence. To directly visualize cargo, organelles were specifically tracked. MitoTracker-stained mitochondria were evident within TNTs (Fig. 3d), consistent with transfer of mitochondria via TNTs^49^. Cellular vesicles were labelled with DiD, which stains lipophilic structures with very low toxicity, does not undergo passive transport and has been used to track cellular vesicles transferred by different ways, including TNTs^15,50–52^. DiD-tracked vesicles were present inside TNTs (Fig. 3e) consistent with our EM data.

**Fig. 4:**
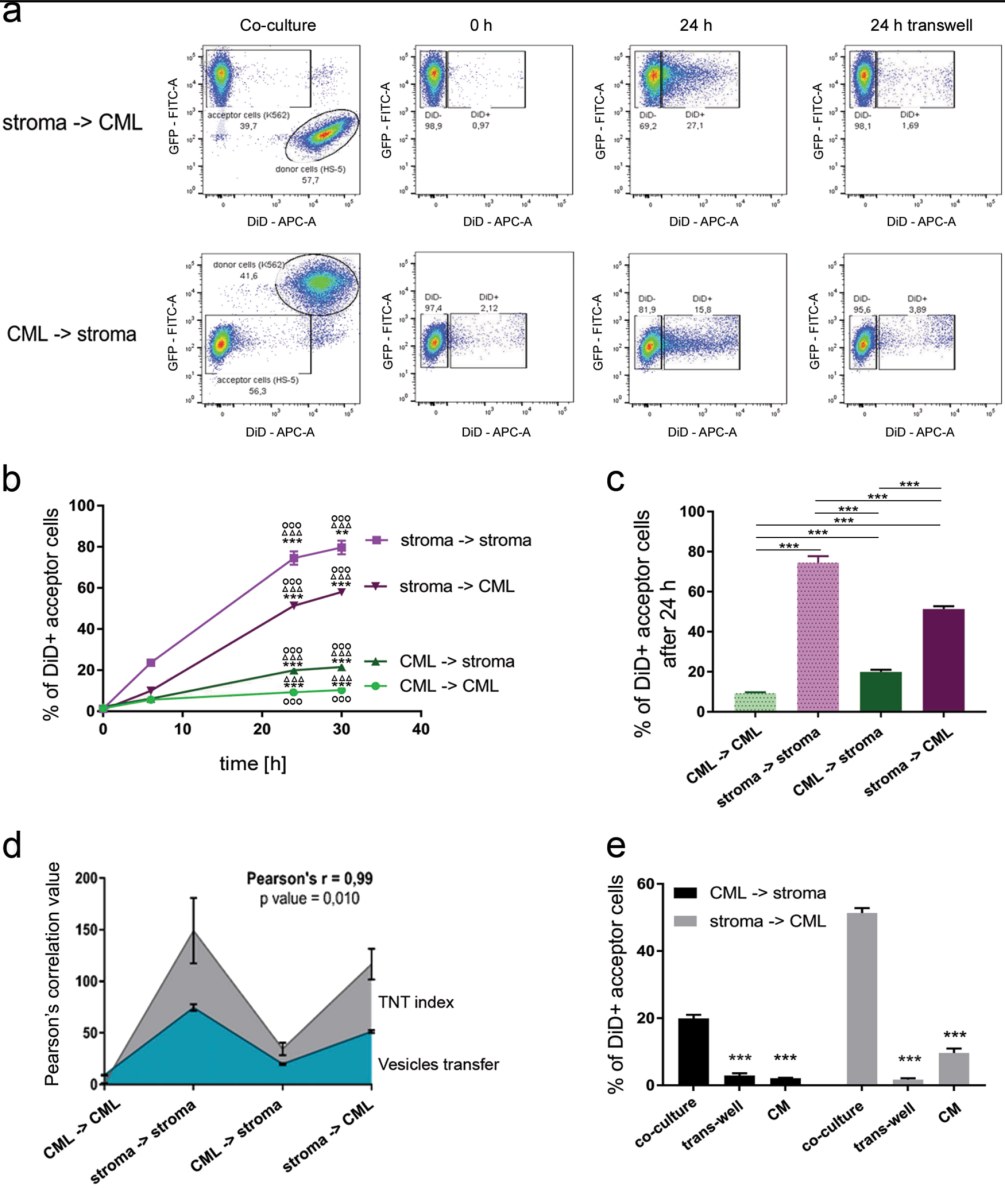
Quantitative analysis of the direct exchange of cellular vesicles between stromal and leukemic cells. **a** Dot plots that present the gating strategy for the flow cytometry experiments on the exchange of cellular vesicles in a co-culture set-up. CML cells expressed cytoplasmic GFP. Donor cells were labeled with DiD for cytoplasmic vesicles. The plots depict the shift in fluorescence in acceptor cells that was caused by the uptake of fluorescently labeled vesicles. The transwell system was used as a control to show that vesicles transfer was directly contact-dependent. **b** Time course of cellular vesicle trafficking in the mono- and co-culture set-ups, quantified by flow cytometry. The percentage of DiD+ acceptor cells is shown. Statistical significance marked in the stroma-stroma transfer was compared to stroma-CML (**), CML-stroma (∆∆∆) and CML-CML (ΟΟΟ) transfer; marked in the stroma-CML transfer was compared to stroma-stroma (***), CML-stroma (∆∆∆) and CML-CML (ΟΟΟ) transfer; marked in the CML-stroma transfer was compared to stroma-stroma (***), stroma-CML (∆∆∆) and CML-CML (ΟΟΟ) transfer; marked in the CML-CML transfer was compared to the stroma-stroma (***), stroma-CML (∆∆∆) and CML-stroma (ΟΟΟ) transfer. **c** Efficiency of cellular vesicle trafficking in the mono- and co-culture set-ups, quantified by flow cytometry and presented as the average percentage of acceptor cells that received fluorescently labeled vesicles that were transferred from donor cells after 24 h of culture. Statistical significance was calculated between indicated values. **d** The Pearson’s correlation calculated for efficiency of vesicles transfer and TNT index. **e** Control experiments for TNT-promoting conditions of the stroma-leukemia vesicles transfer. Donor cells were labeled with DiD for cytoplasmic vesicles. For the control experiments, (i) a transwell system was used to physically separate donor and acceptor cells that shared the same medium, or (ii) acceptor cells were cultured in conditioned medium (CM) that was collected from donor cells, to allow for exchange of secreted but not directly transferred vesicles. The percentage of DiD+ acceptor cells is shown. Statistical significance was calculated to co-culture control. All of the data are expressed as the mean ± SEM of three independent experiments

To assess the efficiency of mitochondria intercellular transport, we adapted flow cytometry (Fig. 4a), previously used to quantify the nanotube-mediated vesicles transfer^14,52^. The TNT-mediated transfer of stromal GFP-expressing mitochondria, (Supplementary Fig. 3a), was confirmed by a transwell, estimated as a percentage of GFP-positive leukemic acceptor cells (Supplementary Fig. 3b) and visualized (Supplementary Fig. 3c).

Next, HS-5 cells possessing DiD-tracked vesicles were co-cultured with CML cells expressing GFP, to distinguish stromal donors from leukemic recipients^15,49–51^. In this case we quantified vesicles accumulated during 24 h of transfer, shown as a percentage of DiD-positive (DiD+) acceptor cells (Fig. 4a). Interestingly, the transfer efficiency depended on the donor cell type (Fig. 4b). In mono-cultures, stromal cells were efficient donors, whereas CML cells were very poor donors (Supplementary Fig. 4, Fig. 4b, c). In co-culture, transfer from stromal to leukemic cells was significantly more efficient (Fig. 4b, c). The Pearson’s correlation of the level of TNT cargo transfer with the counts of TNTs further indicated that TNTs might be responsible for the vesicles trafficking (Fig. 4d).

To exclude that secretion of extracellular vesicles could underlie the transfer observed by flow cytometry, acceptor cells were cultured in the 24 h conditioned medium (CM) of donor cells that were labeled with DiD (Fig. 4e). The transfer of vesicles in CM-treated acceptor cells was substantially lower than the transfer that occurred under co-culture conditions allowing direct cell-cell contact and TNT formation (Fig. 4e, CM). To further assess the role of cell-cell contact, necessary for TNT formation, acceptor cells were physically separated from donor cells by a transwell with 1.0 µM pores which enabled cells to share the same media (containing exosomes and other extracellular vesicles) but excluded the possibility of physical direct contact. Transfer of dye was significantly lower in the transwell conditions compared to co-culture (Fig. 4e).

M-Sec protein has been proposed as a regulator of TNT formation in macrophages^34,53^ and in U2OS osteosarcoma cell line^54^. To test whether M-Sec is a general regulator of TNTs, M-Sec expression was silenced in HS-5 and K562 cells (Supplementary Fig. 5a, b). TNT-mediated vesicle transfer was not affected by M-Sec silencing (Supplementary Fig. 5c). This suggests that M-Sec does not regulate vesicle transfer from stroma to leukemia and indicates that M-Sec function as TNT driver might be cell-type specific. Given that M-Sec depletion could not be used to verify the involvement of TNTs in intercellular cargo transfer, we instead subjected co-cultures to starvation, previously reported to stimulate the formation and activity of TNTs^35^. Indeed, starvation upregulated vesicle trafficking from stroma to leukemia (Supplementary Fig. 5d). Altogether, these data establish that the stroma-to-leukemia intercellular vesicle transfer occurs in TNT-promoting conditions, is cell-to-cell contact-dependent and is not mediated by secreted extracellular vesicles.

### Direct transfer of cellular vesicles from stroma protects CML cells from imatinib-induced apoptosis

**Fig. 5:**
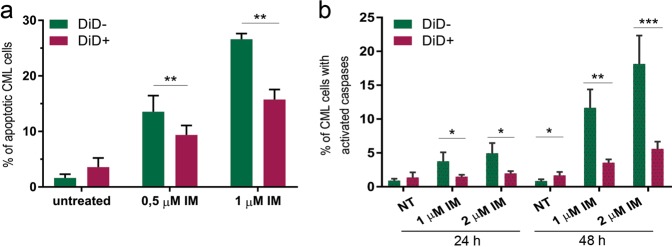
Protection of leukemic cells from imatinib-induced apoptosis by the transported vesicles. **a** Percentage of apoptotic leukemic cells upon treatment with imatinib, analyzed separately for cells that received (DiD+) or did not receive (DiD-) cellular vesicles from stromal cells. **b** Percentage of leukemic cells with active caspases upon treatment with imatinib, analyzed separately for cells that received (DiD+) or did not receive (DiD-) cellular vesicles from stromal cells. Statistical significance was calculated between indicated values. All of the data are expressed as the mean ± SEM of three independent experiments

The TNT-mediated transfer of vesicles has been described previously but crucially, biological consequences of such transfer have not been established. As CML cells are protected from imatinib-driven apoptosis upon co-culture with stromal cells^55,56^ (Supplementary Fig. 6), we addressed whether or not TNT-mediated transfer of vesicles confers this protection. DiD-stained stromal cells were co-cultured with CML cells treated with imatinib. Apoptosis was detected separately in subpopulations of CML acceptor cells, that received (DiD+) or did not (DiD-) receive fluorescent vesicles from stroma. We found that uptake of vesicles from stroma correlated with increased protection against imatinib-driven apoptosis (Fig. 5a), visible as significantly lower percentage of Annexin V-staining in DiD+ CML cells, compared with CML cells negative for vesicles (DiD−). In addition, activity of effector caspases -1, -2, -3, -6, -8, -9, and -10 in imatinib-treated leukemic cells was significantly lower in the DiD+ population (Fig. 5b). On the other hand, mitochondrial transfer was not upregulated after 24 h, and slightly upregulated after 48 h of imatinib treatment, however, the percentage of recipient leukemic cells was still very low (below 4%) indicating that mitochondrial transfer is probably not a crucial rescue mechanism in imatinib response (Supplementary Fig. 6b). Altogether, these data are consistent with direct transfer of cellular vesicles from stromal to leukemic cells being important for acquiring protection from imatinib-induced apoptosis.

### Specific sets of proteins are transported with vesicles from stroma to leukemia

**Fig. 6:**
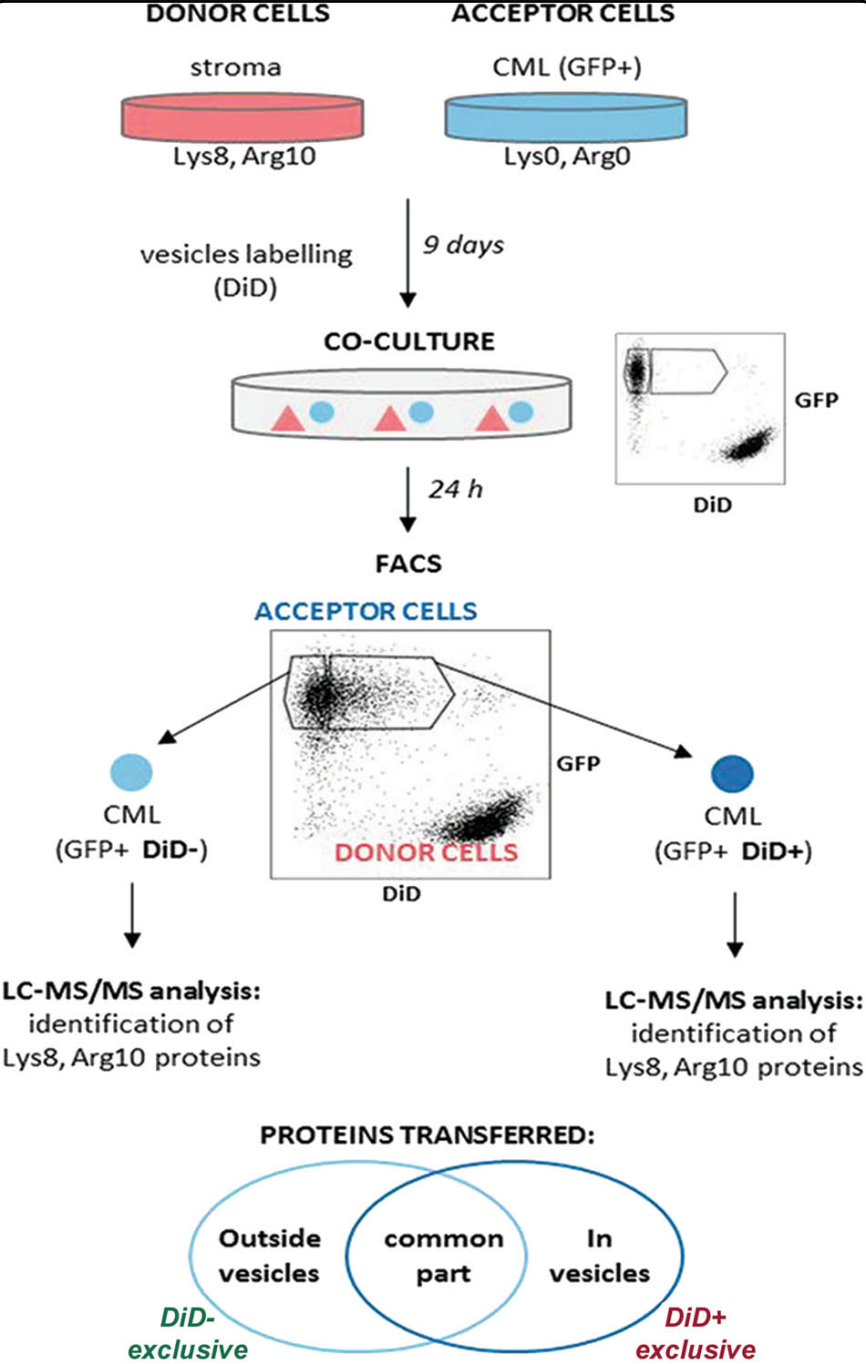
The scheme showing the mass spectrometry (MS)-based trans-SILAC approach performed to identify proteins that are present within stroma-derived vesicles. Donor stromal cells were cultured in media containing heavy isotopologues of Lys and Arg for 9 days to allow complete incorporation of these amino acids into their proteome. They were stained with DiD for cellular vesicles and subjected to co-culture with acceptor CML cells for 24 h. Afterwards two subpopulations of CML cells were sorted using FACS: cells which received (DiD+) or did not receive (DiD-) cellular vesicles from donor stromal cells. In both samples, heavy proteins transferred from donor stromal cells, were identified. Only proteins present exclusively in the DiD+ sample were considered to have been transferred along with the studied vesicles

We hypothesized that the stroma-dependent protection of leukemic cells might result from proteome exchange that occurs together with TNT-dependent transfer of vesicles. To identify proteins which are directly transferred between HS-5 stromal and K562 CML cells together with vesicles, we used mass spectrometry (MS)-based trans-SILAC, combined with fluorescent vesicle tracking with DiD and cell sorting (Fig. 6). Donor stromal cells were cultured in media containing heavy isotopologues of lysine and arginine to allow for >98.5% incorporation (Supplementary Table 2) and additionally stained for vesicles with DiD. Then co-cultured with acceptor cells and followed by sorting of GFP-positive CML cells positive (DiD+) or negative (DiD−) for the stroma-derived vesicles. Liquid chromatography-tandem MS enabled to identify heavy-labeled proteins within the proteome of acceptor cells. These proteins were expressed in donor cells and transferred with vesicles to acceptor cells during co-culture.

**Fig. 7:**
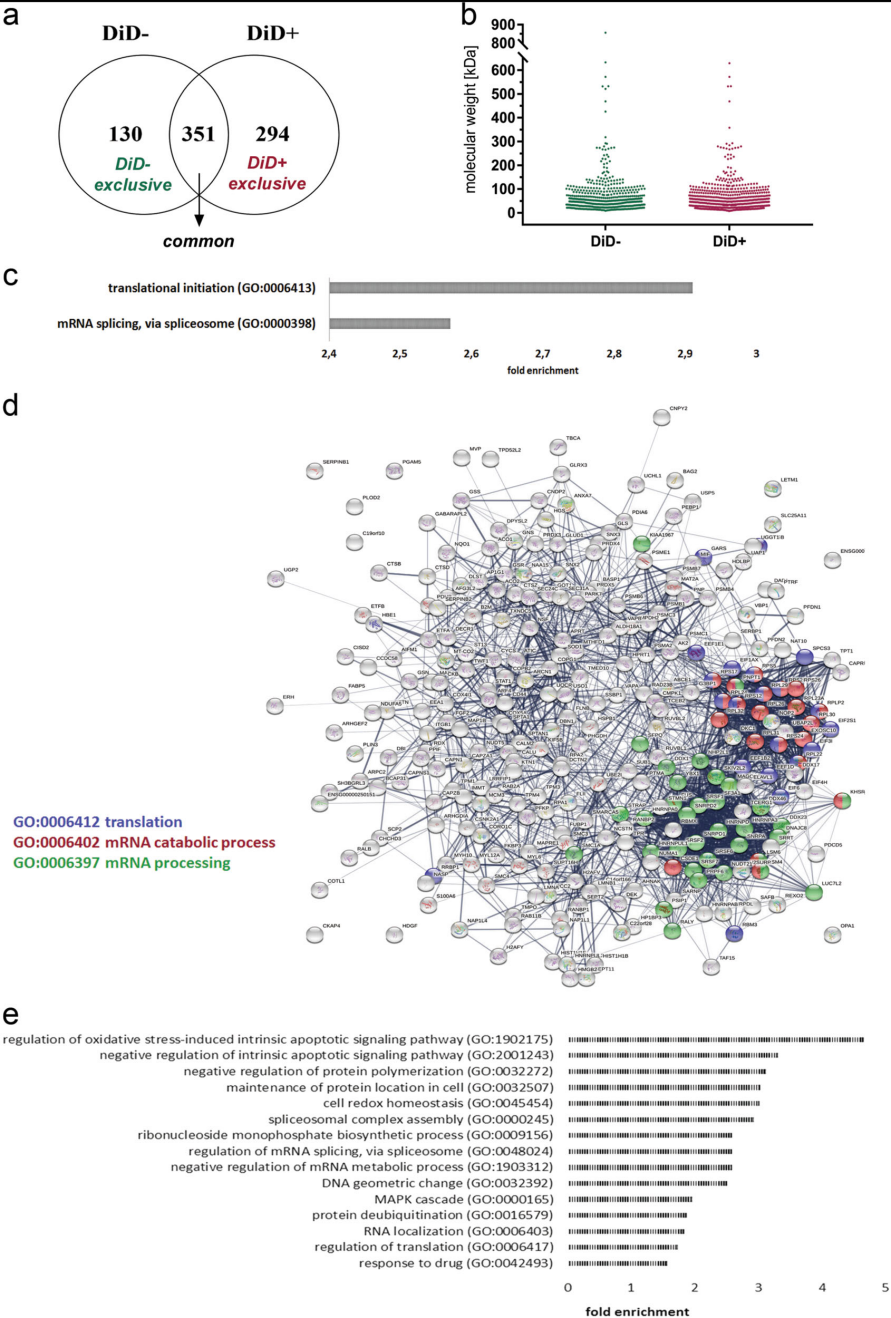
Identification of proteins transported along with cellular vesicles from stromal to leukemic cells. **a** Venn diagram comparison of sets of proteins that were transferred from stromal to CML cells either separately (DiD-) or together with DiD-labeled cellular vesicles (DiD+). **b** Molecular weights of proteins that were transferred from stromal to CML cells separately (DiD-) or together with DiD-labeled cellular vesicles (DiD+). **c** Results of statistical overrepresentation test of the DiD+ exclusive list of proteins (Panther GeneOntology). The list was tested for the enrichment of proteins that are involved in GeneOntology Biological Processes against the reference list of genes of the K562 cell line from the PRIDE database, completed with the list of genes that were identified in the present study. Fisher’s Exact with FDR multiple test correction was used. Only results with FDR ≤ 0.05 are presented. **d** Analysis of functional protein association networks (String software) of the DiD+ exclusive list of proteins. Nodes represent proteins. Edges represent protein-protein associations. The line thickness indicates the strength of data support. PPI enrichment *p* < 1.0e−16. **e** Enriched biological processes Gene Ontology term analysis of proteins that were transferred along with DID-labelled cellular vesicles (DiD+)

We identified 481 donor-derived proteins in the K562 DiD− population and 646 in the K562 DiD+ population (Supplementary Table 3), with 351 proteins in common (Fig. 7a). 294 proteins (DiD+ exclusive) were transferred exclusively to leukemia cells together with DiD-tracked vesicles. Similar molecular weight distribution (Fig. 7b), demonstrated that regardless of the transport mechanism, there was no obvious size restriction.

Statistical overrepresentation analysis against the reference set showed that within the DiD- exclusive group of proteins, no biological processes were enriched, whereas in the DiD+ exclusive group, proteins involved in translation initiation and mRNA splicing were specifically enriched (Fig. 7c). This corresponded to the STRING analysis of DiD+ exclusive proteins, showing a functional protein-protein interaction net (Fig. 7d; PPI enrichment *p* < 1.0e−16). Two major hubs in the network contained proteins involved in mRNA processing and translation or the mRNA catabolic process. These results suggested that cellular vesicles transferred in a TNT-promoting conditions to CML cells, might serve as a source of proteins to modulate gene expression at the level of mRNA processing and translation in CML cells.

Next, the complete DiD+ and DiD− lists of proteins were compared after a statistical overrepresentation test. GeneOntology analysis identified 15 biological processes that were enriched exclusively in the DiD+ sample (Fig. 7e, Supplementary Table 4). Already identified regulation of mRNA splicing, RNA localization, RNA metabolic processes and translation, but also novel ones: negative regulation of apoptotic signaling, cell redox homeostasis, and response to drug, were strongly overrepresented in the DiD+ group (Fig. 7e, Supplementary Table 4). Thus, the proteins transported via TNTs and enriched specifically in leukemic cells which obtained stromal vesicles might specifically promote survival and response to stress conditions. Altogether, our data show the novel mechanism of stroma-provided protection of CML cells from imatinib, involving direct transfer of cellular vesicles and proteins. To our knowledge this is the first evidence indicating the biological role of vesicles transferred from stroma to leukemia via tunneling nanotubes.

## Discussion

The present study provides new insights into the stroma-provided protection of leukemic cells and highlights the important role of a direct cell-to-cell trafficking of cellular vesicles and proteins that can support the cytoprotective and anti-apoptotic responses of leukemic cells; with TNTs likely being involved in the observed exchange.

The presence of leukemic TNTs has been reported previously^37,39,49,57^ although their formation within leukemia microenvironment is not well explored. Here, we present evidence that TNTs formed between stromal and CML cells facilitate transfer of mitochondria, cellular vesicles and proteins. The presence of stroma stimulated leukemic cells to form TNTs, consistent with previous work showing that AML cells isolated from bone marrow formed more TNTs, than cells without stromal component^37^. Leukemia-stroma TNTs were shown also to regulate cytokines secretion^39^ and mediate mitochondrial transfer^49^.

To date, no specific molecular markers of TNTs have been uncovered. Hence, the presence of characteristic morphological features is required to identify TNTs^19,58^. Here, we confirmed that membrane connections formed between leukemic cells and stroma possess features to be classified as TNTs. We excluded the possibility that they are filopodia-like protrusions, or adhesive gap-junctions, previously described in leukemia^59^. We also established that TNT formation occurs between these cell types following “cell dislodgement”^14,60,61^. A similar process has been reported for immune cells^14,15,62^ and cells of other origin^63^.

There is still a limited data describing the 3D ultrastructure of TNTs. Recently, the first Cryo-EM studies of neuronal TNTs were published^18^. Here, we performed CLEM and electron tomography 3D ultrastructure analysis of stromal TNTs. We were able to detect single tubes by CLEM, after single TNT detected by fluorescent microscopy. We also observed thicker TNTs, which were revealed to be bunches of multiple TNTs, as recently found between neurons^18^. Thus, either single TNTs or TNT bunches can be formed within bone marrow stroma.

There is much debate as to whether or not nanotubes may possess open or closed ends and form a synaptic connection. Here, we provide evidence that TNTs can have both: some TNTs seem to have open ends while others can be closed. Similar indications were noticed also for neuronal TNTs^18^. Detection of intercellular transfer of cargo, provided a functional proof for existence of open structures binding two distant cells enabling transfer. We propose that both types representing open- and close-ended TNTs can exist simultaneously. The possibility that TNTs are indeed highly dynamic, open-close structures, cannot be excluded, but needs advanced studies providing the 3D reconstruction of the very same TNT, combined with life imaging studies.

Importantly, no general pharmacological inhibitors of TNT activity have been discovered to date. Hence, the only possibility to provide evidence that the observed cargo transfer is TNT-mediated is to perform indirect experimental verifications. Thus, using different microscopic approaches, as well as the transwell and conditioned medium controls we provided indirect, however complex correlative data, confirming that the stroma to leukemia vesicle transfer, occurs exclusively in TNT-promoting conditions. Such approach has been used by others to imply TNT involvement^24^. Our data showing that M-Sec silencing does not have impact on TNTs formation in leukemic microenvironment are in agreement with similar observations in AML cells^37^. Hence, our data are consistent with vesicle transfer from stromal to leukemic cells being mediated via TNTs.

Crucially, we found that TNT-mediated transfer of vesicles from stroma correlates with the protection of leukemic cells from imatinib-induced apoptosis. Exchange of vesicles has been previously described, but mostly as a proxy to studying TNT formation, rather than to address specific functional consequences^13,52^. It was proposed previously that receiving mitochondria in a TNT-dependent way, may be a potent mechanism of survival^49^. Also, TNT-dependent secretion of proleukemic cytokines^39^, transfer of oncogenes^33^ and miRNAs^26^ can not be neglected as part of the tumor-stromal cross-talk and potent regulators of oncogenic signaling in acceptor cells. Our data indicate that TNT-mediated transfer of cellular vesicles from stroma is a novel cytoprotective mechanism participating in leukemia resistance.

Interestingly, we found that functional sets of proteins can be transferred together with vesicles from stromal to leukemic cells. The transfer of single proteins via TNTs has been reported^24,64^, however, we demonstrated that whole sets of proteins can be exchanged between cells. Identified proteins can form functional networks to regulate biological processes that are important for cytoprotection. We propose that they either directly help CML cells cope with drug response/apoptosis or indirectly remodel their proteome at the levels of transcription and translation to promote adaptation and survival. As a general finding, our data support the hypothesis of intercellular proteome exchange^65,66^.

Altogether, our data are in line with others, indicating TNTs as promising therapeutic target in cancer. Despite detection of different inhibitory compounds, the specific TNT inhibitors are still not developed^20^. Different strategies to inhibit TNTs formation and function are proposed, including those inhibiting microfilament formation^67^, mTOR pathway or vesicular transport controlled by the small Rab GTPases^68^. This data rather suggest that there are multiple TNT formation pathways, which are selectively expressed depending on cellular context^20^. So, even if targeting of TNTs is promising and opens up novel possibilities for therapies, challenges still remain.

In summary, we described the ultrastructure of stromal TNTs and present evidence for TNT-transferred vesicles in the protection of leukemic cells. Thus, TNT-mediated cross-talk may be crucial to the stroma-provided imatinib resistance, and if so, then TNT-mediated communication would be a potential therapeutic target in the treatment of leukemia.

## Supplementary information

Supplementary Figure Legends

Supplementary Figure 1

Supplementary Figure 2

Supplementary Figure 3

Supplementary Figure 4

Supplementary Figure 5

Supplementary Figure 6

Supplementary Movie S1

Supplementary Movie S2

Supplementary Movie S3

Supplementary Table 1

Supplementary Table 2

Supplementary Table 3

Supplementary Table 4

DECLARATION OF CONTRIBUTIONS TO ARTICLE
