# Supplementary material for: Tunneling nanotube-mediated intercellular vesicle and protein transfer in the stroma-provided imatinib resistance in chronic myeloid leukemia cells
Source: Cell Death Dis. 2019 Oct 28;10(11):817. doi: 10.1038/s41419-019-2045-8 (PMC6817823; doi:10.1038/s41419-019-2045-8)
Supplement: Supplementary file 1 — Supplementary Figure Legends [file 41419_2019_2045_MOESM1_ESM.docx]

**Supplementary Figure Legends**

**Supplementary Figure 1.** Cytoskeletal components within TNTs. Representative images showing the actin (green) and microtubule (red) present inside a TNTs that interconnected stromal cells. Blue indicates nuclei. The bottom panel shows the overlay.

**Supplementary Figure 2.** Participation of plasma membranes of stromal cells and CML cells in heterotypic TNT formation, quantified by confocal microscopy after 48 h of co-culture. **a**. Representative images (left panels) and statistical analysis (right diagram) of heterotypic TNTs that were formed by plasma membranes of both stromal cells and CML cells (upper panel) or exclusively by plasma membranes of CML cells (middle panel) or stromal cells (lower panel). CML cells were transfected by nucleofection with a GPI-GFP-encoding plasmid, FACS sorted, and subjected to co-culture with stromal cells. All cell membranes were stained with WGA-Alexa Fluor 647 directly before imaging. Scale bars = 10 μm. **b**. Average lengths of heterotypic TNTs that depended on the origin of the plasma membrane that constituted a given TNT. All of the data are expressed as the mean ± SEM of three independent experiments.

**Supplementary Figure 3.** Analysis of TNT-mediated mitochondria transfer from stromal to leukemic cells. **a**. Representative picture showing HS-5 stromal cells expressing GFP-tagged mitochondria and stained with DAPI to visualize nucleus. K562 leukemic were cell tracked with proliferation eFluor 570 dye (red). Scale bar = 10 μm. **b**. Flow cytometry analysis of mitochondria transfer from HS-5 donors to K562 leukemic recipient cells after 24 hour co-culture as well as in the trans-well system control. The percentage of GFP-positive leukemic recipient cells is shown. Data are expressed as the mean ± SEM of three independent experiments at least. **c**. Leukemic cell (green arrow) showing GFP signal representing the mitochondria transferred after 24h co-culture with HS-5 stromal cells expressing GFP-tagged mitochondria. K562 leukemic cells were tracked with proliferation eFluor 570 dye (red). Scale bar = 10 μm.

**Supplementary Figure 4.** Dot plots that present the gating strategy for the flow cytometry experiments on the exchange of cellular vesicles in a mono-culture set-up. CML cells expressed cytoplasmic GFP. Donor cells were labeled with DiD for cytoplasmic vesicles. The plots depict the shift in fluorescence in acceptor cells that was caused by the uptake of fluorescently labeled vesicles. The trans-well system was used as a control to show that vesicles transfer was directly contact-dependent.

**Supplementary Figure 5.** Role of M-Sec protein in TNTs activity and transfer of cellular vesicles between stromal and leukemic cells. M-Sec expression levels in HS-5 (**a**) and K562 cells (**b**) transfected with negative or M-Sec shRNA. Tubulin was used as a loading control. Marker size is indicated. **c**. Efficiency of vesicles transfer from stromal to leukemic cells upon M-Sec silencing. HS-5 stromal donor were stained with DiD to track vesicles, leukemic K562 recipients were tracked with WGA-AF 488. Transfer was estimated after 24 hour co-culture by flow cytometry as a percentage of AF+,DiD+ leukemic recipient cells. Trans-well system was used to block TNTs formation and to verify TNT-dependent transfer.

**Supplementary Figure 6. a.** Percentage of apoptotic leukemic cells cultured alone or in co-culture with stromal cells, upon treatment with 0,5 or 1 μM imatinib. Percentage of Annexin V-positive leukemic cells was calculated. Data are expressed as the mean ± SEM of three independent experiments. **b**. Transfer of GFP-expressing mitochondria from stromal to leukemic cells after 24 and 48 hours of imatinib treatment calculated by flow cytometry. Percentage of GFP-positive acceptoc leukemic cells is presented. All of the data are expressed as the mean ± SEM of three independent experiments.
