# Supplementary figures and images for: Tunneling nanotube-mediated intercellular vesicle and protein transfer in the stroma-provided imatinib resistance in chronic myeloid leukemia cells
Source: Cell Death Dis. 2019 Oct 28;10(11):817. doi: 10.1038/s41419-019-2045-8 (PMC6817823; doi:10.1038/s41419-019-2045-8)

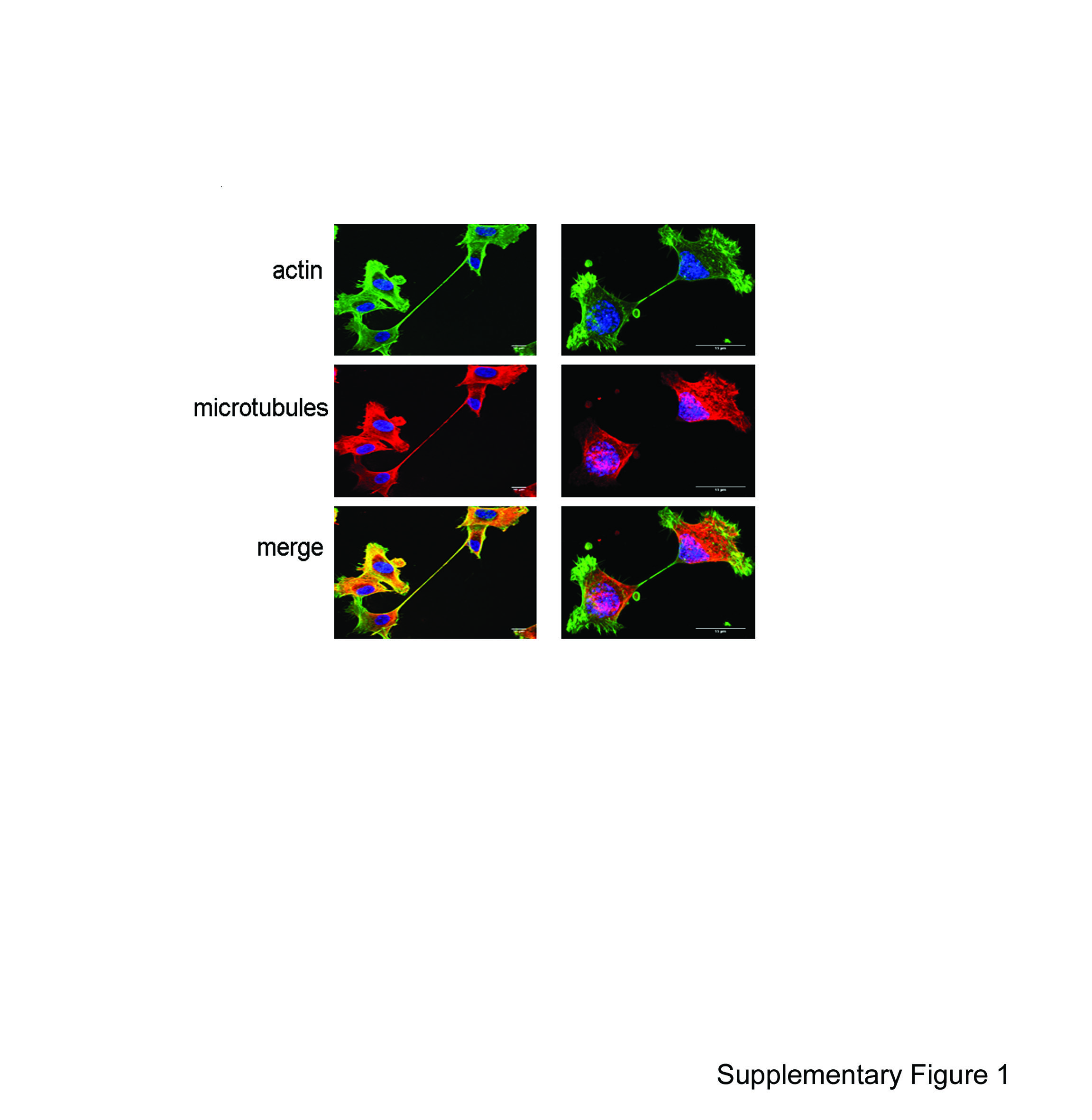

Supplement: Supplementary file 2 — Supplementary Figure 1 [file 41419_2019_2045_MOESM2_ESM.tif]

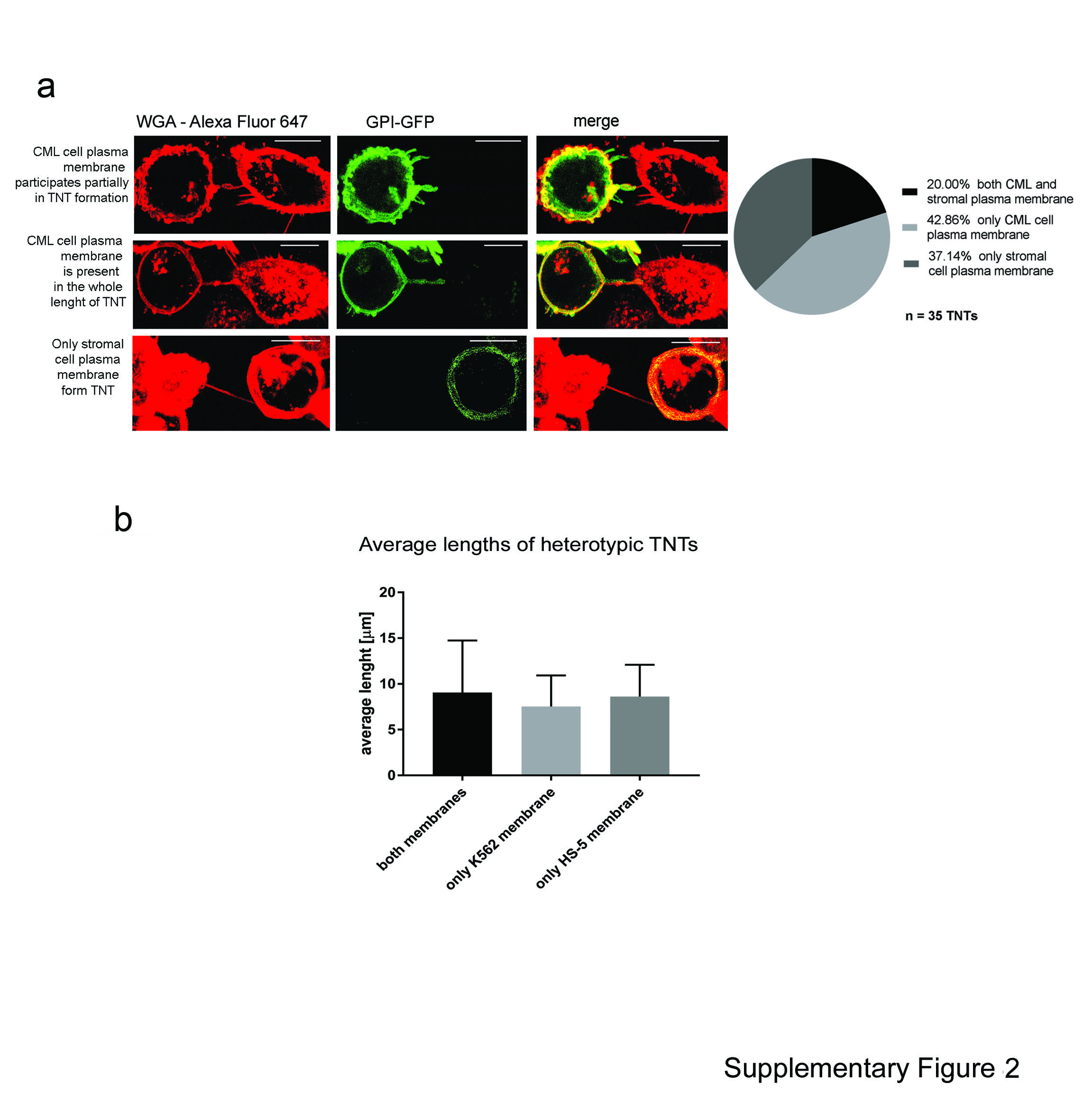

Supplement: Supplementary file 3 — Supplementary Figure 2 [file 41419_2019_2045_MOESM3_ESM.tif]

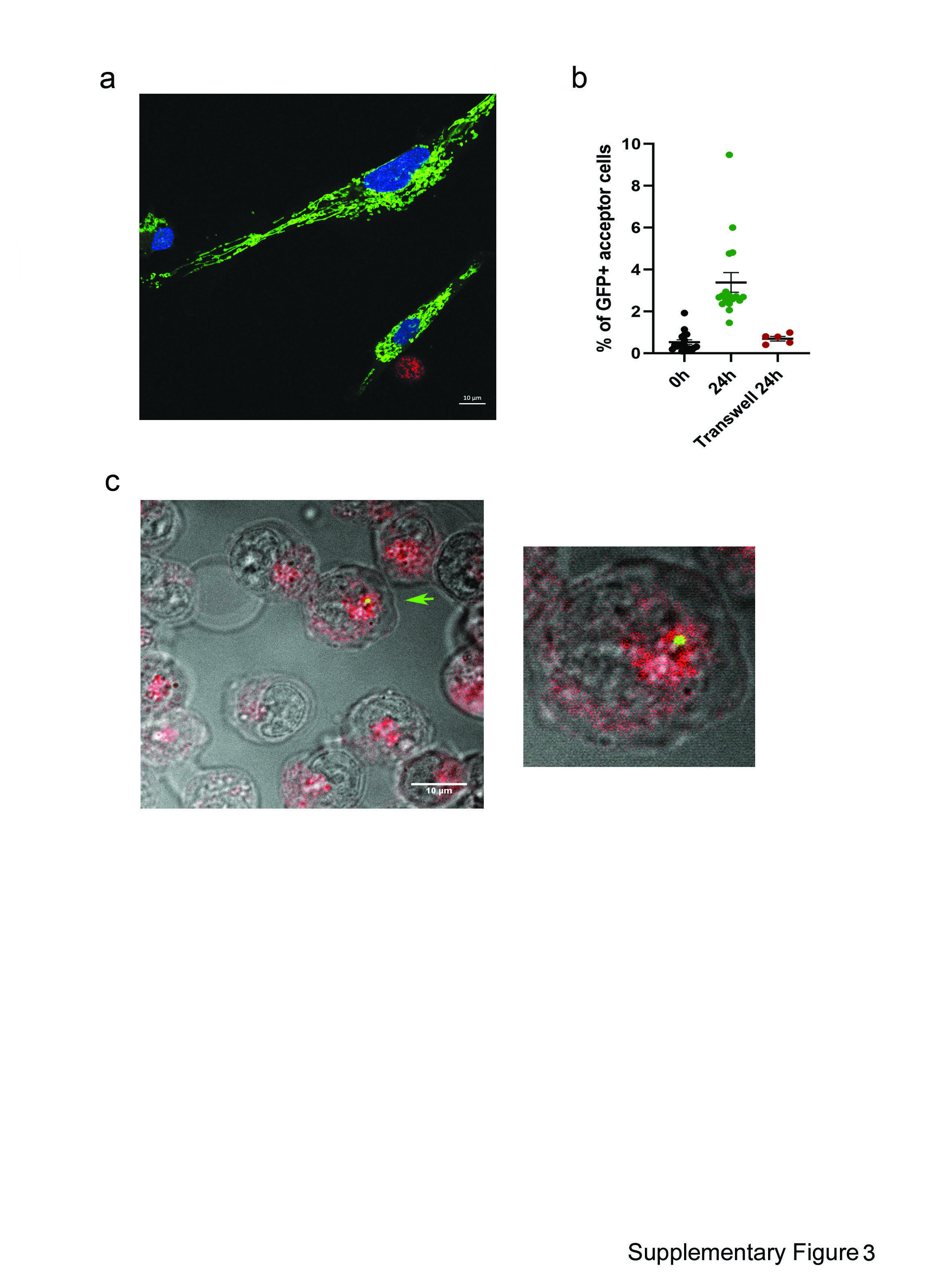

Supplement: Supplementary file 4 — Supplementary Figure 3 [file 41419_2019_2045_MOESM4_ESM.tif]

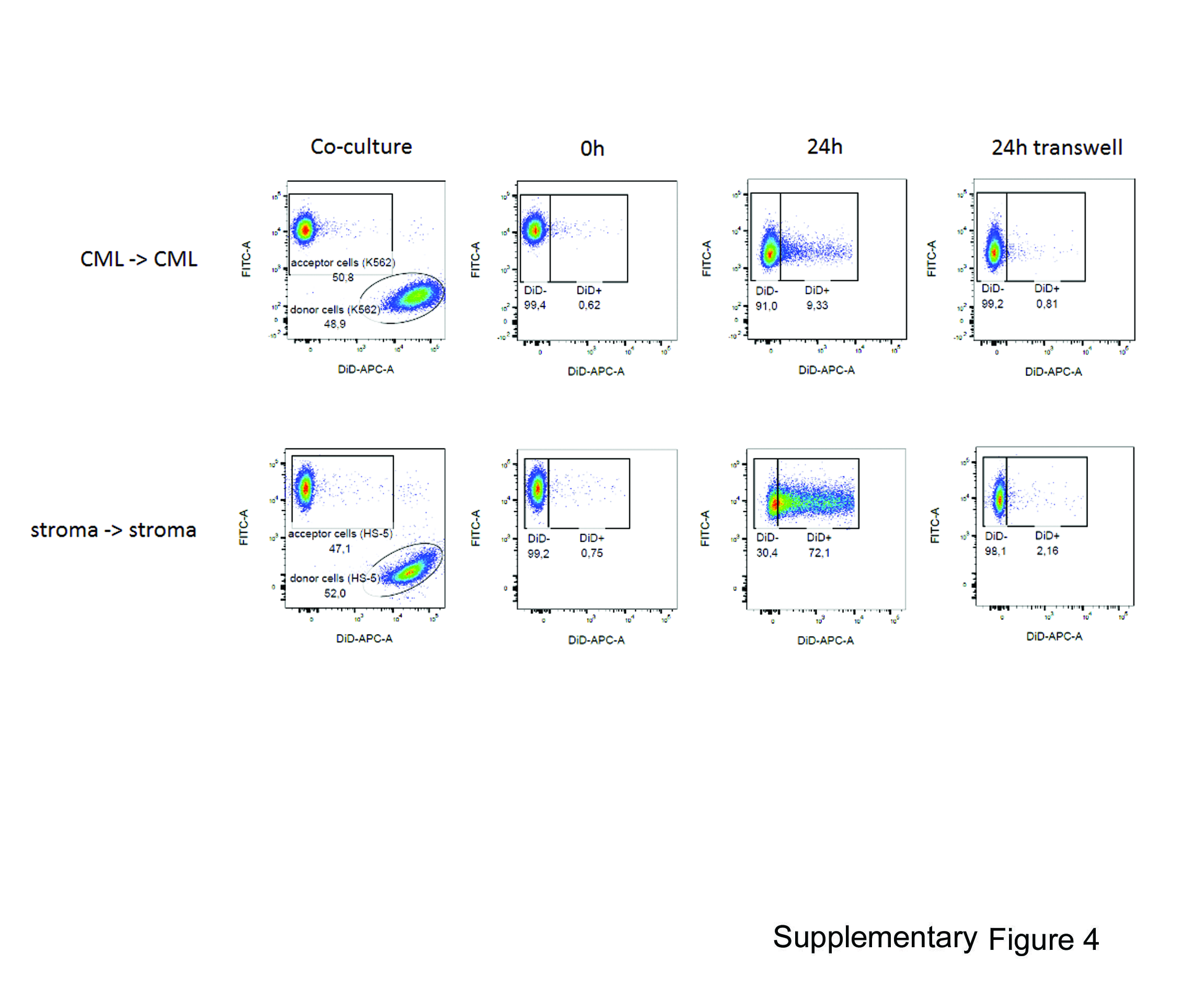

Supplement: Supplementary file 5 — Supplementary Figure 4 [file 41419_2019_2045_MOESM5_ESM.tif]

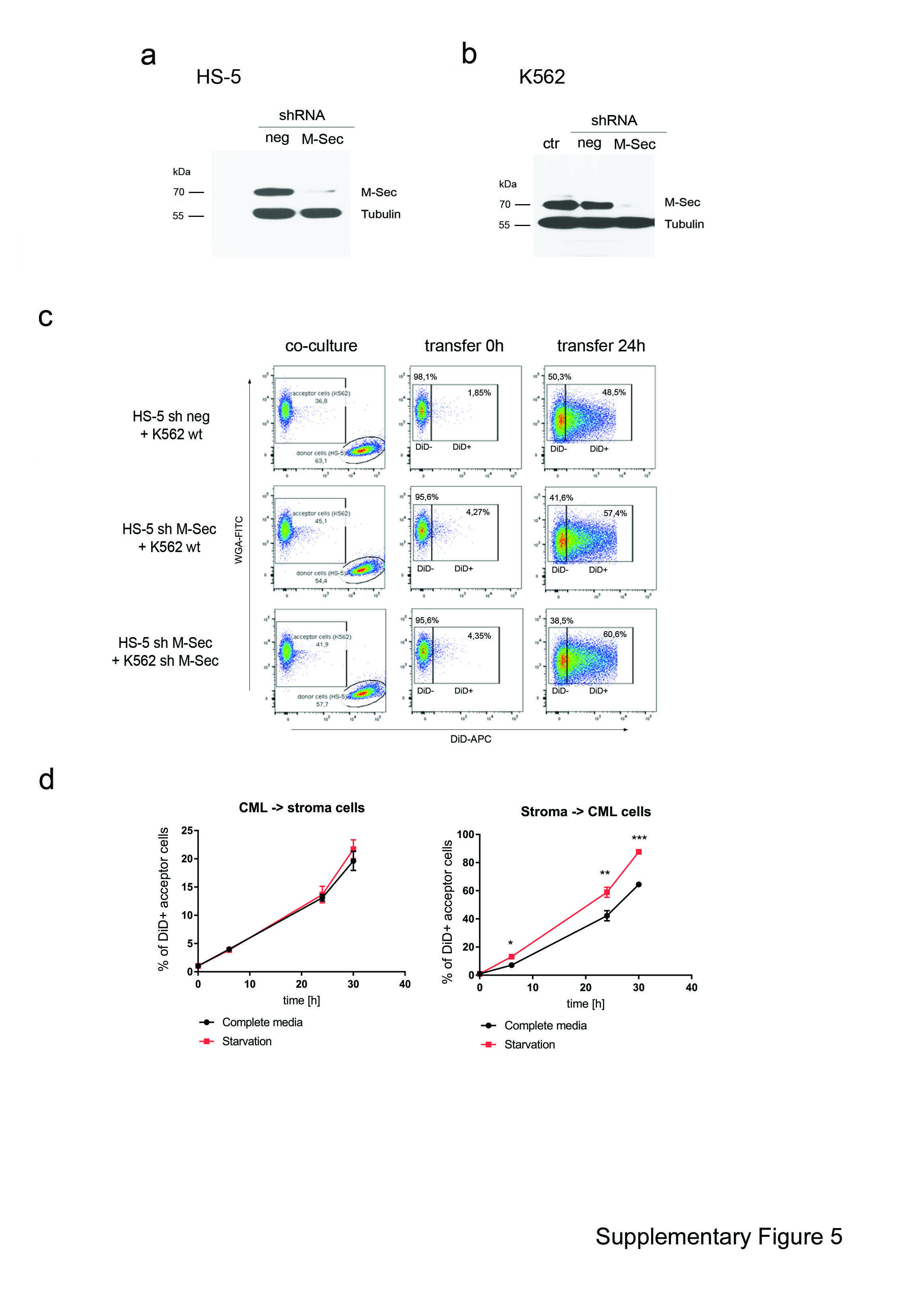

Supplement: Supplementary file 6 — Supplementary Figure 5 [file 41419_2019_2045_MOESM6_ESM.tif]

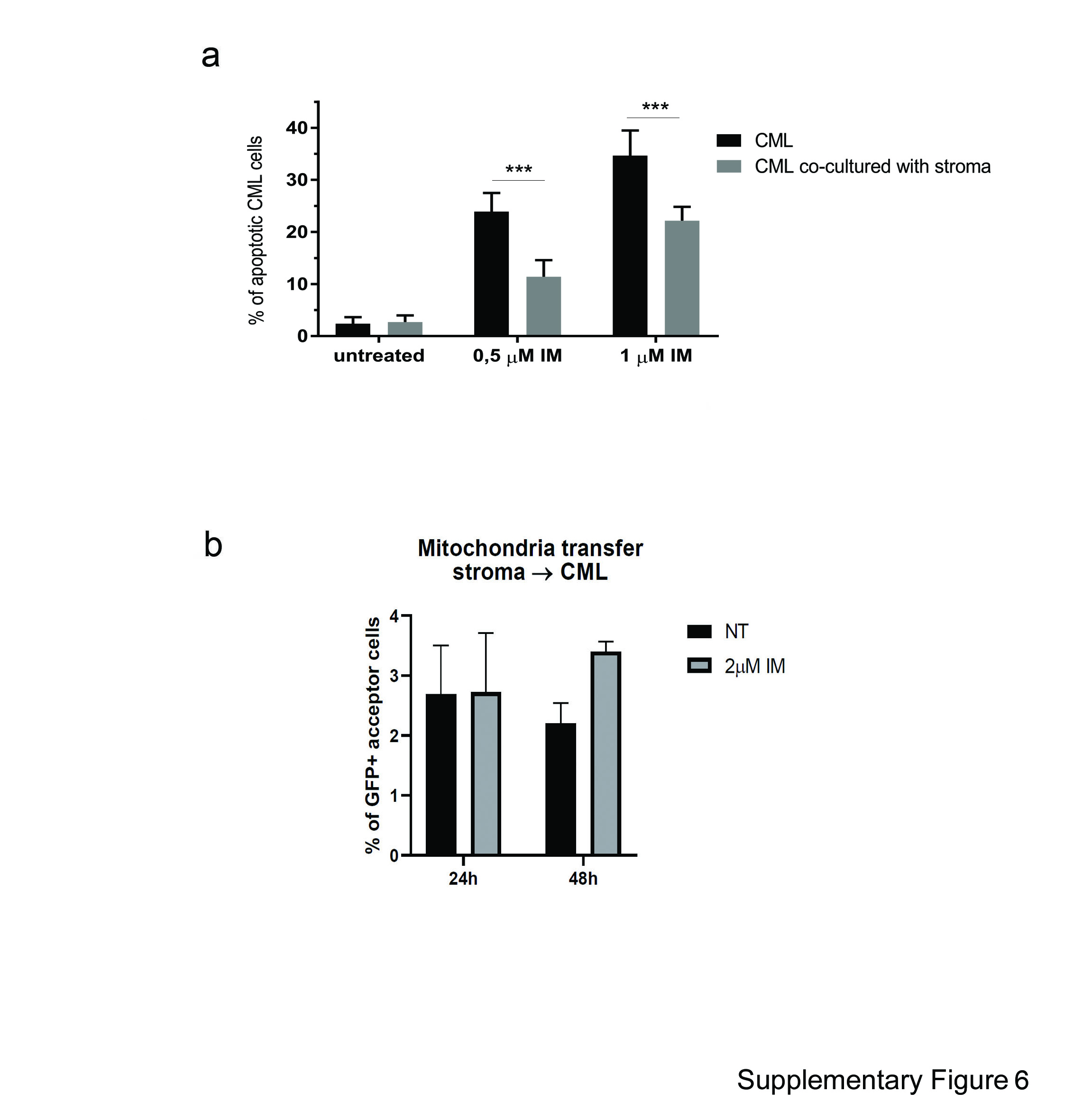

Supplement: Supplementary file 7 — Supplementary Figure 6 [file 41419_2019_2045_MOESM7_ESM.tif]
