# Supplementary material for: Tunneling nanotube-mediated intercellular vesicle and protein transfer in the stroma-provided imatinib resistance in chronic myeloid leukemia cells
Source: Cell Death Dis. 2019 Oct 28;10(11):817. doi: 10.1038/s41419-019-2045-8 (PMC6817823; doi:10.1038/s41419-019-2045-8)
Supplement: Supplementary file 15 — DECLARATION OF CONTRIBUTIONS TO ARTICLE [file 41419_2019_2045_MOESM15_ESM.pdf]

DECLARATION OF CONTRIBUTIONS TO ARTICLE **ADMC**

Manuscript Number:	Journal Name:	
CDDIS-19-1766R	*Cell Death & Differentiation*	(the 'Journal')

Proposed Title of the Contribution:

Tunneling nanotube-mediated intercellular vesicle and protein transfer in the stroma-provided imatinib resistance in chronic myeloid leukemia cells (the 'Contribution)

Author(s):

Marta Kolba , Wioleta Dudka , Monika Zaręba-Kozioł , Agata Kominek , Paolo Ronchi , Laura Turos , Piotr Chroscicki , Jakub Wlodarczyk , Yannick Schwab , Agata Klejman , Dominik Cysewski , Katja Srpan , Daniel Davis, Katarzyna Piwocka (the 'Authors')

For all *CDD* articles, each person named as an author in the published version must be able to show he or she has contributed substantially to the article.

Authorship credit should be based on 1) substantial contributions to conception and design, acquisition of data, or analysis and interpretation of data; 2) drafting the article or revising it critically for important intellectual content; and 3) final approval of the version to be published. Authors should meet conditions 1, 2 and 3.

Any person who cannot be shown to have made a substantial contribution to the article cannot be listed as an author in the final version. The name of any person who is deemed to have made a minor contribution can, however, appear in the Acknowledgments section of the article.

Please complete the table below to indicate the contributions of all named authors to the manuscript.

Author Full Name:	Specification of Contribution to the Manuscript:
Marta D. Kolba	Substantially contributed in designing and performing most of the experiments, analyzed the data, participated in data interpretation, drafted the manuscript and assembled Figures. Finally approaved the manuscript.
Wioleta Dudka	Designed and performed CLEM experiments under supervision of PR and YS. Performed SEM experiments together with MDK. Analysed and discussed the data, participated in data interpretation and rafting the manuscript. Assembled Figures. Finally approaved the manuscript.
Monika Zaręba-Kozioł	Contributed in designing MS experiments, prepared the samples for MS and analyzed MS data. Participated in data interpretation and drafting the manuscript. Critically revised the manuscript. and assembled MS Figures and Tables. Finally approaved the manuscript.
Agata Kominek	Contributed in planning and performing cell sorting and flow cytometry analyses. Drafted the manuscript. Finally approaved the manuscript.
Paolo Ronchi	Substantialy contributed in conception, designing of CLEM experiments, and interpretation of the data. Critically discussed and revised the manuscript. Finally approaved the manuscript.
Laura Turos	Participated in designing, performing and acqusition of the caspase activity test and M-Sec silencing experiments under supervision of MDK. Analysed the data and assembled the Figures. Finally approaved the manuscript.
Piotr Chroscicki	Contributed in designing and generation of HS-5 cells with GFP-mitochondria and studied mitochondrial transfer, analysed , discussed and interpreted the data. Drafted the manuscript and assembled the Figures. Finally approaved the manuscript.
Jakub Wlodarczyk	JW contributed in designing the mass spectrometry experiments and supervised MZ-K. Discussed the data, critically discussed and revised the manuscript. Finally approaved the manuscript.
Yannick Schwab	Substantialy contributed in conception, designing and supervision of CLEM experiments, discussed and participated in interpretation of the data. Critically discussed and revised the manuscript. Finally approaved the manuscript.
Agata Klejman	Designed and performed viral transduction to silence M-Sec, drafted the manuscript. Finally approvaed the manuscript.
Dominik Cysewski	Substantially participated in analysis and discussion of MS data and data interpretation. Participated in tables assessment. Finally approaved the manuscript.
Katja Srpan	Involved in performing of microscopy experiments and imaging of TNTs together with MDK. Discussed the data. Finally approaved the manuscript.
Daniel M. Davis	Contributed in planning experiments, supervised KS . Participated in data discussion and interpretation. Critically discussed and revised the manuscript. Finally approaved the manuscript.

DECLARATION OF CONTRIBUTIONS TO ARTICLE **ADMC**

Manuscript Number:	Journal Name:	
CDDIS-19-1766-T CDDIS-19-1766R	*Cell Death & Differentiation*	(the 'Journal')

Proposed Title of the Contribution:

Tunneling nanotube-mediated intercellular vesicle and protein transfer in the stroma-provided imatinib resistance in chronic myeloid leukemia cells (the 'Contribution)

Author(s):

Marta Kolba , Wioleta Dudka , Monika Zaręba-Kozioł , Agata Kominek , Paolo Ronchi , Laura Turos , Piotr Chroscicki , Jakub Wlodarczyk , Yannick Schwab , Agata Klejman , Dominik Cysewski , Katja Srpan , Daniel Davis, Katarzyna Piwocka (the 'Authors')

For all *CDD* articles, each person named as an author in the published version must be able to show he or she has contributed substantially to the article.

Authorship credit should be based on 1) substantial contributions to conception and design, acquisition of data, or analysis and interpretation of data; 2) drafting the article or revising it critically for important intellectual content; and 3) final approval of the version to be published. Authors should meet conditions 1, 2 and 3.

Any person who cannot be shown to have made a substantial contribution to the article cannot be listed as an author in the final version. The name of any person who is deemed to have made a minor contribution can, however, appear in the Acknowledgments section of the article.

Please complete the table below to indicate the contributions of all named authors to the manuscript.

Author Full Name:	Specification of Contribution to the Manuscript:
Katarzyna Piwocka	Conception and design of the studies. Contributed in data interpretation and discussion and final figures preparation. Writed and discussedthe manuscript. Finally approaved the manuscript.

Please complete the table below to indicate the contributions of all named authors to the figures.

Figure 1:

MD. Kolba and K. Piwocka - conception and desing, data analysis and interpretation; MD Kolba and W. Dudka - performing experiments. MD. Kolba and K. Piwocka - Figure preparation.

Figure 2:

W. Dudka, P. Ronchi, Y. Schwab and K. Piwocka - conception and desing; W. Dudka, P. Ronchi - performimg experiments and data analysis; W. Dudka, P. Ronchi, Y. Schwab, K. Piwocka - data interpretation and discussion; W. Dudka, K. Piwocka - Figure preparation

Figure 3:

MD. Kolba and K. Piwocka - conception and desing, data analysis and interpretation; MD Kolba - performing experiments. MD. Kolba and K. Piwocka - Figure preparation .

Figure 4:

MD. Kolba, K. Piwocka - conception and design; MD Kolba, A. Kominek - design of flow cytometry protocols; MD Kolba, A. Kominek - performing flow cytometry experiments and data analysis; MD Kolba, K. Piwocka - data interpretation; MD. Kolba and K. Piwocka - Figure preparation.

Figure 5:

MD. Kolba, L. Turos, K. Piwocka - planning and desing; MD. Kolba, L. Turos - performing experiments and data analysis; MD. Kolba, L. Turos, K. Piwocka - data interpretation and Figure preparation.

Figure 6:

MD. Kolba, K. Piwocka - conception and design; MD. Kolba - Figure preparation.

Signed for and on behalf of the Author(s):	Print Name:	Date:
[signature]	Katarzyna Piwocka	01.10.2019

Please complete the table below to indicate the contributions of all named authors to the figures.

Figure 1:

Figure 7
MD. Kolba, K. Piwocka - studies conception; MD.Kolba, J. Wlodarczyk, D. Cysewski and K. Piwocka - MS experiments design; MD.Kolba and M. Zaręba-Kozioł - performing experiments; MD.Kolba, M. Zaręba-Kozioł , D. Cysewski - data analysis; MD.Kolba, M. Zaręba-Kozioł , J. Wlodarczyk, K. Piwocka - data interpretation. MD. Kolba, M. Zaręba-Kozioł , D. Cysewski , K. Piwocka - Figure preparation

Figure 2:

Supplementary Figure 1
MD. Kolba and K. Piwocka - conception and desing, data analysis and interpretation; MD Kolba - performing experiments; MD. Kolba and K. Piwocka - Figure preparation.

Figure 3:

Supplementary Figure 2
MD. Kolba and K. Piwocka - conception and desing, data analysis and interpretation; MD Kolba - performing experiments; MD. Kolba and K. Piwocka - Figure preparation.

Figure 4:

Supplementary Figure 3
P. Chroscicki and K. Piwocka - conception and desing; P. Chroscicki - performing the experiments and data analysis; P. Chroscicki and K. Piwocka - data interpretation; P. Chroscicki and K. Piwocka - Figure preparation.

Figure 5:

Supplementary Figure 4
MD. Kolba, K. Piwocka - conception and design; MD Kolba, A. Kominek - design of flow cytometry protocols and experiment; MD Kolba, A. Kominek - performing flow cytometry experiments and data analysis; MD Kolba, K. Piwocka - data interpretation; MD. Kolba and K. Piwocka - Figure preparation.

Figure 6:

Supplementary Figure 5
MD. Kolba, K. Piwocka - conception and design; MD. Kolba, A. Klejman, L. Turos - performing experiments and data analysis; MD. Kolba, L. Turos, K. Piwocka - data interpretation; MD. Kolba, L. Turos and K. Piwocka - Figure preparation.

Signed for and on behalf of the Author(s):	Print Name:	Date:
[signature]	Katarzyna Piwocka	01.10.2019

Please complete the table below to indicate the contributions of all named authors to the figures.

Figure 1:

Supplementary Figure 6
MD. Kolba, L. Turos, P. Chroscicki, K. Piwocka - conception and design of experiments; MD. Kolba, L. Turos, P. Chroscicki - performing experiments and data analysis; MD. Kolba, P. Chroscicki, K. Piwocka - data interpretation; MD. Kolba, L. Turos, P. Chroscicki, K. Piwocka - Figure preparation

Figure 2:

Supplementary Movie S1
MD. Kolba and K. Piwocka - conception of experiments; MD. Kolba - acquisition of data; MD. Kolba and K. Piwocka - data interpretation

Figure 3:

Supplementary Movie S2
MD. Kolba and K. Piwocka - conception of experiments; MD. Kolba - acquisition of data; MD. Kolba and K. Piwocka - data interpretation

Figure 4:

Supplementary Movie S3
MD. Kolba and K. Piwocka - conception of experiments; MD. Kolba - acquisition of data; MD. Kolba and K. Piwocka - data interpretation

Figure 5:

Supplementary Table 1
MD. Kolba, M. Zaręba-Kozioł, D. Cysewski, J. Włodarczyk and K. Piwocka - conception and design; M. Zaręba-Kozioł, D. Cysewski - data analysis and Table preparation

Figure 6:

Supplementary Table 2
MD. Kolba, M. Zaręba-Kozioł, D. Cysewski and K. Piwocka - conception and design; M. Zaręba-Kozioł, D. Cysewski - data analysis; MD. Kolba - Table preparation

Signed for and on behalf of the Author(s):	Print Name:	Date:
[signature]	Katarzyna Piwocka	01.10.2019

Please complete the table below to indicate the contributions of all named authors to the figures.

Figure 1:

Supplementary Table 3
MD. Kolba, M. Zaręba-Kozioł, D. Cysewski and K. Piwocka - conception and design; M. Zaręba-Kozioł, D. Cysewski - data analysis, MD. Kolba, M. Zaręba-Kozioł - Table preparation

Figure 2:

Supplementary Table 4
Supplementary Table 1
MD. Kolba, M. Zaręba-Kozioł, D. Cysewski, J. Włodarczyk and K. Piwocka - conception and design; M. Zaręba-Kozioł, D. Cysewski - data analysis; MD. Kolba and M. Zaręba-Kozioł - Table preparation

Figure 3:

Figure 4:

Figure 5:

Figure 6:

Signed for and on behalf of the Author(s):	Print Name:	Date:
[signature]	Katarzyna Piwocka	01.10.2019
